# A Case Report of Acute Intermittent Porphyria Mimicking Autoimmune Encephalitis

**DOI:** 10.7759/cureus.106583

**Published:** 2026-04-07

**Authors:** Harwindar Kumar, Maira Salam

**Affiliations:** 1 Neurology, Buner Medical Center, Buner, PAK; 2 Acute Medicine, Queen Elizabeth Hospital, University Hospitals Birmingham NHS Foundation Trust, Birmingham, GBR

**Keywords:** acute porphyria, autoimmune encephalitis, encephalopathy, hematological disorders, seizures

## Abstract

A 21-year-old female patient had recurrent visits to the ED with chest pain managed conservatively, then developed recurrent episodes of seizures and became encephalopathic. She eventually landed in the intensive care unit with extensive investigations to confirm or rule out infective and noninfective etiologies for encephalitis. Her cerebrospinal fluid examination was unremarkable, including autoimmune encephalitis screening. The MRI brain had juxtacortical and in places cortical signal change, with a possibility of an encephalitic process going on. Furthermore, influenza type B was detected on blood screening, and the EEG showed generalized slow-wave discharges. She had dilated pupils, raised blood pressure, and low sodium levels, which pointed toward autonomic instability. Keeping in view autonomic instability, new onset seizures, preceded chest pain, and encephalopathic EEG, she was screened for porphyria and diagnosed with the condition.

## Introduction

Acute intermittent porphyria (AIP) is a rare autosomal dominant metabolic disorder of heme biosynthesis caused by deficiency of the enzyme porphobilinogen (PBG) deaminase (hydroxymethylbilane synthase). It is a multisystem disorder with a prevalence of 5-10 per million [1]. This enzymatic defect leads to the accumulation of neurotoxic heme precursors, primarily δ-aminolevulinic acid (ALA) and PBG, particularly during physiological stress such as infection, fasting, hormonal changes, or exposure to certain medications that induce hepatic ALA synthase. These accumulated intermediates exert neurotoxic effects through mechanisms including oxidative stress, impaired mitochondrial energy metabolism, and interference with GABAergic neurotransmission, resulting in dysfunction of the peripheral nervous system, autonomic nervous system, and central nervous system (CNS).

Clinically, AIP typically presents with acute attacks characterized by severe, poorly localized abdominal pain accompanied by nausea, vomiting, constipation, or ileus. Abdominal imaging is often unremarkable. Prominent autonomic features include tachycardia, hypertension, sweating, and urinary retention due to autonomic neuropathy. Neurological presentation of AIP is as common as 10%-40% in acute episodes. Neurological manifestations may include peripheral motor neuropathy with proximal weakness, sensory disturbances, cranial nerve involvement, and, in severe cases, respiratory muscle weakness or ascending paralysis resembling Guillain-Barré syndrome. CNS involvement produces psychiatric and encephalopathic symptoms such as anxiety, agitation, insomnia, hallucinations, psychosis, confusion, and seizures. Seizures can happen in 5% of people with AIP [2]. These may be worsened by associated metabolic abnormalities, particularly hyponatremia caused by the syndrome of inappropriate antidiuretic hormone secretion.

AIP might be underdiagnosed, and most patients who present with almost similar features are diagnosed with autoimmune encephalitis. Because of the combination of psychiatric symptoms, seizures, cognitive disturbance, and autonomic instability, AIP can closely mimic autoimmune encephalitis, especially forms such as anti-N-methyl-D-aspartate receptor encephalitis, which also present with acute psychiatric and neurological features. This overlap creates significant diagnostic challenges, as patients may initially present to gastroenterology, neurology, or psychiatry services. The absence of cutaneous photosensitivity, as seen in other porphyrias, can further obscure the diagnosis.

However, several distinguishing features help differentiate AIP from autoimmune encephalitis, including the presence of severe unexplained abdominal pain, prominent autonomic and visceral symptoms, recurrent attacks triggered by medications or metabolic stress, and characteristic biochemical abnormalities such as markedly elevated urinary ALA and PBG during acute episodes. Additionally, cerebrospinal fluid (CSF) and neuroimaging are typically normal or nonspecific in AIP, while autoimmune encephalitis often demonstrates inflammatory CSF changes or neuronal autoantibodies. Recognition of these key differences is essential because prompt diagnosis of AIP allows targeted treatment with intravenous hemin and carbohydrate loading, which suppresses hepatic ALA synthase and prevents progression of neurological damage. The management of the two conditions differs; early diagnosis and prompt treatment can be lifesaving.

## Case presentation

A previously well woman in her early 20s, with no past medical history and normal baseline functioning, initially presented several times with what appeared to be a straightforward flu-like illness, complaining of right-sided chest pain and a dry cough. She was treated conservatively for costochondritis and later given antibiotics when imaging showed a left lower lobe consolidation. However, her clinical course took an abrupt and unexpected turn when, within a few days, she developed recurrent seizures. Although she recovered to a normal level of consciousness between the episodes, the sudden onset of seizures prompted admission to intensive care with a presumed diagnosis of meningoencephalitis. She was started on intravenous acyclovir, antibiotics, and levetiracetam. A positive test for influenza B seemed to offer a unifying explanation for her initial symptoms. When CSF microscopy, culture, and viral PCR returned negative, antimicrobial therapy was appropriately discontinued.

However, she continued to deteriorate, which was not consistent with the original diagnosis. Despite control of her seizures, she became increasingly confused, agitated, and unresponsive to commands. At the bedside, there were subtle but important clues toward an alternative process. She was persistently hypertensive, tachycardic, and unusually restless. She had dilated pupils with sluggish responses. She did not have any motor weakness and was moving all four limbs. She could localize pain, and there was no focal neurological deficit or signs of meningism. A key detail only emerged in retrospect; her family reported that she had been experiencing unexplained high blood pressure for several days before admission.

Investigations continued to be inconclusive. EEG ruled out ongoing seizure activity. CSF microscopy, Gram staining, culture, and viral PCR were negative. Autoimmune encephalitis antibody screening in CSF and serum was negative. Autoimmune/vasculitic screening was also negative.

**Figure 1:**
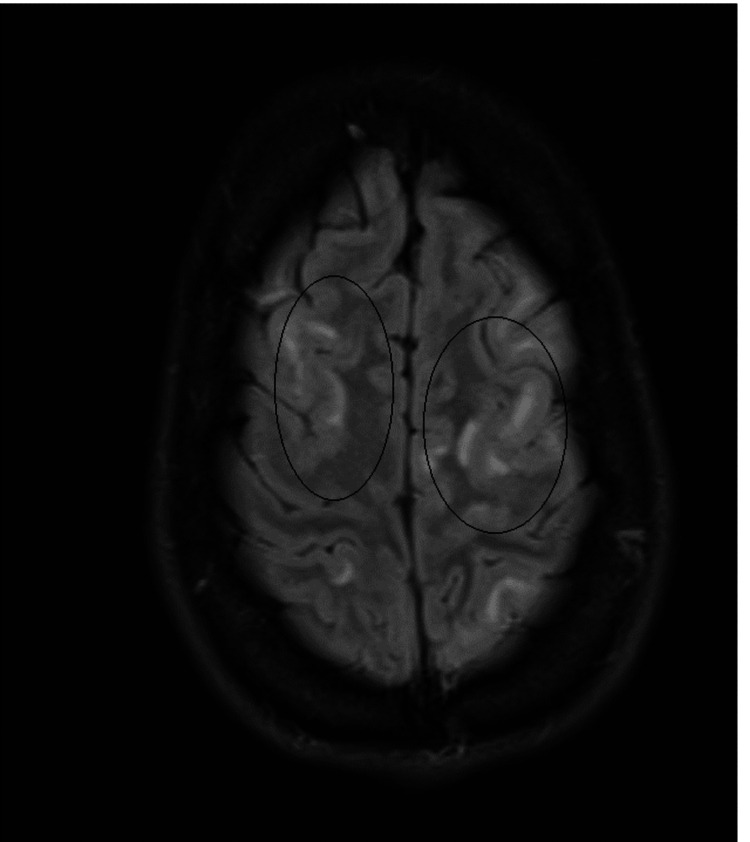
T2-FLAIR sequence axial section at a level near the vertex. This image shows widespread juxtacortical and cortical signal changes. At other levels (not shown in this image), there was also partial effacement of sulcal spaces, particularly in the left temporal region FLAIR: fluid-attenuated inversion recovery

**Figure 2:**
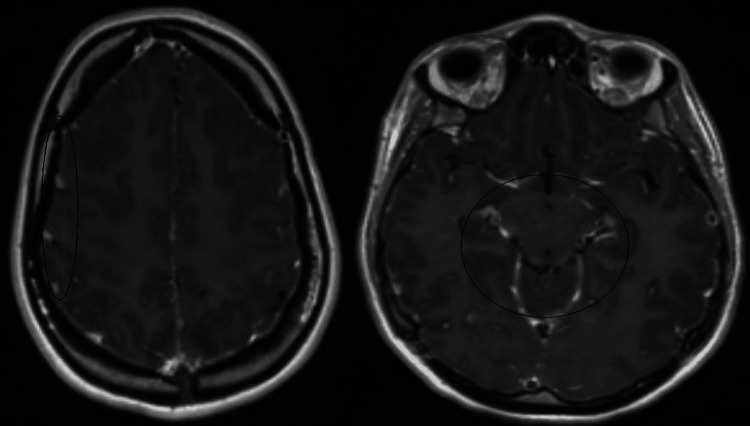
Postgadolinium contrast T1-weighted MRI head axial section at the level near the vertex (left) and postgadolinium contrast T1-weighted MRI head axial section at the level of the midbrain (right). Prominent enhancement within the sulcal spaces of both cerebral hemispheres, likely vascular in nature, although leptomeningeal enhancement cannot be entirely excluded. No definite cranial nerve enhancement

MRI brain with contrast revealed widespread juxtacortical and, in places, cortical signal changes without clear diffusion restriction. Partial effacement of sulcal spaces was noted, particularly in the left temporal region. Prominent enhancement within the sulcal spaces of both cerebral hemispheres and over the brainstem surface was observed following intravenous gadolinium administration, likely vascular in nature, although leptomeningeal enhancement could not be entirely excluded. No definite cranial nerve enhancement was seen. Further patchy signal changes were noted within the cerebrum (left > right). These imaging appearances were suggestive of an encephalitic process (Figures 1, 2). Although the MRI was abnormal, it did not clearly point to a specific diagnosis. Alongside this, her sodium level, initially normal, fell rapidly to 117 mmol/L over three days.

At this point, the pattern becomes easier to recognize in hindsight. The combination of worsening neuropsychiatric symptoms, seizures, clear autonomic instability (with hypertension and tachycardia), significant hyponatremia, and a negative standard workup in a young patient raised a strong suspicion for AIP. In this case, it was only after these elements were considered holistically that porphyria was suggested. Biochemical testing for AIP was positive, confirming the diagnosis, as shown in Table 1. Treatment with intravenous hemin led to a striking recovery, and she was able to be stepped down from intensive care and was eventually discharged home.

**Table 1:** Marked elevation of heme precursors ALA and PBG, along with increased levels of other porphyrins, confirming the diagnosis of acute intermittent porphyria ALA: aminolevulinic acid; PBG: porphobilinogen

Parameter	Result	Normal range	Interpretation
Total porphyrins (urine)	198	<35	↑ Increased porphyrin production
ALA	46	0-3	↑↑ Markedly elevated → acute porphyria
PBG	60	0-1.5	↑↑ Diagnostic of acute porphyria
Uroporphyrin I	202 nmol/L	-	↑ Elevated
Uroporphyrin III	63 nmol/L	-	↑ Elevated
Total uroporphyrin	265	<24	↑ Significant elevation
Heptacarboxylate porphyrin	10	<4	↑ Mild elevation
Coproporphyrin I	61	-	↑ Elevated
Coproporphyrin III	462	-	↑↑ Predominant rise
Total coproporphyrin	523	<115	↑ Marked elevation
Lead level	0.18	<0.48	Normal → excludes lead toxicity

The key learning point from this case lies in recognizing when the initial diagnosis no longer adequately explains the patient’s constellation of symptoms. Here, the persistence and progression of symptoms despite appropriate treatment, combined with negative investigations, signaled the need to reconsider the diagnosis. The emergence of autonomic dysfunction, alongside hyponatremia and neuropsychiatric decline, was the critical clue. When these features are viewed together rather than in isolation, they point to AIP, a rare but treatable condition in which early diagnosis can make a profound difference to outcome.

## Discussion

New-onset seizure followed by encephalopathy is a common neurological presentation of many disorders. Some of these disorders are limited to the CNS, while others are systemic disorders with neurological manifestations. Encephalitis is the most common of these disorders, and most of the patients presenting with new-onset seizures and encephalopathy have encephalitis. Encephalitis may have infective or autoimmune etiologies. However, there are other rare diseases, including porphyria, which also need to be considered, especially if there is accompanying autonomic instability and hyponatremia.

Encephalitis refers to a generalized inflammation of the brain parenchyma. It can be broadly divided into infective and noninfective etiologies. Viruses are the most common causes of infectious etiology [3], while autoimmune phenomena are mainly responsible for noninfectious etiology. Viral encephalitis must be ruled out in patients presenting with fever, seizures, and altered consciousness, as delayed diagnosis might be fatal with irreversible brain damage [4]. After ruling out metabolic abnormalities on blood tests and viral encephalitis on CSF testing, autoimmune encephalitis is considered in the differential diagnosis. CSF in autoimmune encephalitis usually shows pleocytosis. However, it might also have normal cells and proteins in 27% of people [5]. An antibody panel for autoimmune encephalitis is tested in both serum and CSF. However, negative antibodies for autoimmune encephalitis screening do not rule out the disease [6]. Neither CSF pleocytosis nor positive screening antibodies are always present in autoimmune encephalitis. This is the reason why patients are sometimes clinically diagnosed with seronegative autoimmune encephalitis and started on immunosuppression. However, if this diagnosis is wrong and the patients have another rare disease that is missed, then starting immunosuppression may cause harm rather than benefit.

Porphyria is a disorder of the heme biosynthesis pathway [7]. It is inherited in an autosomal dominant manner [8]. Neurological complications of porphyria can involve the peripheral nervous system, CNS, and autonomic nervous system [9]. It is a rare disorder, and neurological involvement is even more infrequent. Peripheral nerve involvement is the most common neurological complication of acute porphyria [7]. Peripheral nervous system involvement can present as symmetrical axonal polyneuropathy. However, asymmetrical presentation is not uncommon and may precede the symmetrical presentation. Involvement of the autonomic nervous system may lead to hypertension, resting tachycardia, abdominal pain, dilated pupils, and low sodium levels [10]. CNS involvement can manifest as acute encephalopathy leading to seizures, altered consciousness, headache, and behavior problems [11]. MRI of the head during an acute attack of porphyria can show cortical and subcortical lesions that may show mild enhancement with contrast [12]. Clinical and MRI changes in acute porphyria are usually related to "posterior reversible encephalopathy syndrome" [13]. It is proposed that a decrease in cerebral nitric oxide production during an acute attack, coupled with heme deficiency, can lead to hypertension and cerebral vasospasm [14]. The differences and similarities between autoimmune encephalitis and AIP have been summarized in Table 2.

**Table 2:** Key differences and similarities between autoimmune encephalitis and AIP when presenting with seizures and altered consciousness NMDA: N-methyl-D-aspartate receptor; LGI1: leucine-rich-glioma-inactivated-1; CASPR2: contactin-associated-protein-like-2; PBG: porphobilinogen deaminase; ALA: aminolevulinic acid; PRES: posterior reversible encephalopathy syndrome; CSF: cerebrospinal fluid; FLAIR: fluid-attenuated inversion recovery; RCVS: reversible cerebral vasoconstriction syndrome; IVIg: intravenous immunoglobulin; IV: intravenous

Feature	Autoimmune encephalitis	AIP
Nature of condition	Immune system mistakenly attacks the brain	Inherited metabolic disorder affecting heme synthesis
Primary cause	Autoantibodies targeting neuronal surface antigens (e.g., NMDA receptor, LGI1, CASPR2) or intracellular antigens)	Deficiency in PBG deaminase, leading to accumulation of porphyrin precursors (ALA and PBG)
Seizure/altered consciousness presentation	Rapid onset seizures (focal or generalized), confusion, behavioral changes, psychosis, memory deficits, and catatonia. May progress to nonconvulsive status epilepticus	Acute neurological attacks with seizures (often generalized tonic-clonic), confusion, altered mental status, and hallucinations. PRES is a recognized complication
Associated neurological symptoms	Movement disorders (dyskinesias), autonomic instability, psychiatric symptoms, and speech difficulties	Peripheral neuropathy, autonomic dysfunction (abdominal pain, tachycardia, hypertension), anxiety, insomnia, and psychiatric symptoms
Key diagnostic markers	CSF: autoantibodies, pleocytosis, and elevated protein. MRI: T2/FLAIR hyperintensities. EEG: epileptiform activity and extreme delta brush	Urine/blood: elevated ALA and PBG. MRI: may show PRES or RCVS. EEG: generalized slowing or epileptiform discharges
Precipitating factors	Often idiopathic; may be triggered by infections, tumors (paraneoplastic), or vaccinations	Medications (barbiturates, sulfonamides), alcohol, fasting, stress, and hormonal changes (luteal phase)
Treatment focus	Immunosuppression: corticosteroids, IVIg, plasma exchange. Targeted antibody therapy. Antiepileptic drugs for seizures	Heme therapy: IV hematin or heme arginate. Carbohydrate loading for milder attacks. Analgesia and trigger avoidance
Prognosis	Variable: ranges from full recovery to significant long-term deficits, depending on antibody type and treatment speed	Generally good with prompt treatment. Recurrent attacks may cause chronic neurological damage
Key distinguishing feature	Immune-mediated brain attack; specific autoantibodies and inflammatory CSF changes are hallmarks	Metabolic neurotoxicity from porphyrin precursor accumulation; abdominal pain and identifiable triggers are hallmarks

In our patient, a negative antibody panel for autoimmune encephalitis, the development of autonomic instability, the decline of serum sodium level, and ongoing neuropsychiatric decline despite a normal metabolic profile and normal CSF results shifted the differential toward AIP. This was confirmed by biochemical testing for porphyrins, following which treatment with intravenous hemin was initiated. She responded to treatment and improved dramatically. Therefore, she was stepped down to the general ward and eventually discharged from the hospital, with plans for outpatient follow-up by the metabolic medicine team once she returned to her baseline.

## Conclusions

Fever, seizures, and altered consciousness are common and nonspecific presentations of many disorders that may directly or indirectly involve the CNS. Encephalitis is often overdiagnosed in such clinical scenarios, and rare but treatable metabolic conditions like porphyria may be overlooked, potentially leading to fatal outcomes. Clinicians should remain open-minded when assessing patients presenting with new-onset seizures and altered consciousness. The presence of autonomic instability and worsening hyponatremia should specifically alert the clinician toward the possibility of AIP. It should be noted that EEG findings of generalized slowing and MRI findings of cortical/juxtacortical signal change, mild effacement of sulcal spaces, enhancement of sulcal spaces, and surface of brainstem after gadolinium administration might also be present in AIP. Porphyrin testing should be done in such cases, as elevated porphyrin levels would confirm the diagnosis. Delays in diagnosis or management resulting from a preoccupied focus on a single presumed etiology may cause a permanent disability.
